# The spectrum of nasal colonization: frequency and resistant patterns in diabetes versus non-diabetes population

**DOI:** 10.1186/s12866-026-04751-z

**Published:** 2026-02-04

**Authors:** Maryam Rabeh, Samaneh Shahrokh, Mojtaba Akbari, Najmeh Ansari, Mansour Siavash, Maryam Yazdi

**Affiliations:** 1 Department of Microbiology, Faculty of Biological Sciences and Technology, Shahid Ashrafi Esfahani University, Isfahan, Iran; 2 Isfahan Endocrine and Metabolism Research Center, Isfahan University of Medical Sciences, Khorram Street, Isfahan, Iran; 3 Clinical Microbiology Laboratory, Imam Hussein Hospital, Isfahan, Iran; 4 Child Growth and Development Research Center, Research Institute for Primordial Prevention of Non-Communicable Diseases, Isfahan University of Medical Sciences, Isfahan, Iran

**Keywords:** Antimicrobial resistance, Colonization, Diabetes, Enterobacteriaceae, Nasal, *Staphylococcus aureus*

## Abstract

**Background:**

The nasal cavity serves as a primary contact site and is a common location for colonization by symbiotic, opportunistic, and potentially pathogenic bacteria. Diabetic patients are more susceptible to colonization by opportunistic microorganisms due to impaired immune function, altered normal flora, and increased exposure to healthcare. This study aimed to investigate the nasal colonization of Gram-positive (*Staphylococcus aureus*) and Gram-negative (*Enterobacteriaceae*) bacteria in diabetic and non-diabetic individuals, assessing phenotypic traits including antibiotic resistance and biofilm production, as well as investigating the presence of resistant genes.

**Materials and methods:**

In this cross-sectional study, nasal swabs were collected from 150 diabetic and 150 non-diabetic individuals. Isolates were identified and evaluated phenotypically (Antibiotic resistance using the disk diffusion method and biofilm formation by the microtiter plate method) and genotypically (resistance genes including *mecA*, *bla*_CTX_, *bla*_SHV_, and *bla*_TEM_) by PCR.

**Results:**

The rate of *S. aureus* colonization was higher in diabetics (18.7%) than in non-diabetics (12.7%) and MRSA colonization was significantly higher in diabetics (8% vs. 1.3%). High antibiotic resistance was not observed except for tetracycline (nearly 50%) in *S. aureus* isolates from both groups. There was no statistically significant difference in the occurrence of MDR *S. aureus* between the diabetic (32.1%) and non-diabetic (31.6%) groups. *Enterobacteriaceae* colonization was 3.3% in diabetics and 7.3% in non-diabetics. Although none were phenotypically ESBL-positive, *bla*_CTX_, *bla*_TEM_, *and bla*_SHV_ genes were present in about 40% of the isolates.

**Conclusion:**

Nasal MRSA colonization was more common among diabetic patients than non-diabetics. The findings of this study highlight the need for ongoing monitoring of nasal colonization of MRSA in different populations and settings, which may lead to the development of effective preventive and therapeutic strategies to control infections caused by nasal colonization.

**Supplementary Information:**

The online version contains supplementary material available at 10.1186/s12866-026-04751-z.

## Background

The nasal cavity and nasopharynx are among the body’s first points of contact with the external environment, and as part of the upper respiratory tract, they play a crucial role in the initial defense against pathogens. This area hosts diverse microbial communities that include symbiotic, opportunistic, and sometimes potential pathogens [1]. The composition of the nasal microbiota is influenced by several factors, including age, environmental conditions, the individual’s immune status, and underlying diseases. Patients with diabetes are at a higher risk of colonization and infection with opportunistic microorganisms due to impaired immune function, changes in the composition of the body’s natural flora, and increased exposure to healthcare facilities. Among the nasal colonizing bacteria, *S. aureus* is the focus of clinical research, particularly due to its ability to transform from an asymptomatic colonizer to an invasive pathogen [2].

*Staphylococcus aureus* is one of the most important human pathogens and a leading cause of severe infections associated with high mortality, morbidity, and significant healthcare costs. Its wide array of virulence factors enables it to cause multiple clinical manifestations such as bacteremia, endocarditis, osteomyelitis, and infections of the skin, soft tissues, bones, lungs, and medical devices [3]. According to a systematic review and meta-analysis, mortality rates from *S. aureus* bacteremia are 18.1% at one month, 27.0% at three months, and 30.2% at one year (4). *S. aureus* is also frequently isolated from diabetic foot infections (DFIs) worldwide. A recent systematic review conducted in Iran found that *S. aureus* represents 24.29% of bacterial isolates from diabetic foot infections (DFIs), with 55% of these strains being methicillin-resistant (MRSA) [4]. Moreover, up to 30% of the general population may carry *S. aureus* in their nasal passages. Widespread use of antibiotics has led to the emergence of resistant strains such as MRSA, which has significantly increased morbidity and mortality in both hospital and community settings. Occasionally, nasal colonization by *S. aureus* may progress to serious opportunistic infections [5].

On the other hand, the Enterobacteriaceae family includes a large group of gram-negative, aerobic-facultatively anaerobic bacilli that are naturally present in the digestive tract of humans and animals and are considered to be the most important causes of hospital and community-acquired infections [6]. *Enterobacteriaceae* are of great importance in human pathogenesis due to their ability to cause a wide range of intestinal and extraintestinal infections. Many members of this family can cause gastroenteritis, diarrhea, dysentery, and food poisoning. And others, by leaving the intestines and entering various tissues of the body, can lead to urinary tract infections, sepsis, respiratory infections, and wound infections, especially in people with weakened immune systems. Also, the presence of numerous virulence factors, such as endotoxin (LPS), toxins, capsule, and adhesion factors, allows these bacteria to establish themselves well in the host body and cause severe diseases. On the other hand, the ability to be transmitted through contaminated water and food has led to Enterobacteriaceae playing an important role in infectious epidemics and public health, and they are recognized as one of the most important pathogenic bacterial families in humans [7].

Although Gram-negative bacteria are not usually recognized as the dominant nasal flora, recent studies have shown that Gram-negative bacilli such as *Klebsiella spp*., *Escherichia coli*,* Enterobacter spp*., and *Pseudomonas spp*. can also lead to nasal colonization in certain circumstances, including hospitalized patients, people with diabetes, or those with a history of antibiotic use. The importance of these bacteria is doubled when they are associated with multiple drug resistance, especially the production of extended-spectrum beta-lactamases (ESBL) [8]. Considering the increasing prevalence of diabetes worldwide and the key role of nasal bacteria in nosocomial and community-acquired infections, the study of colonization patterns of Gram-positive and Gram-negative bacteria in the nose of diabetic patients is of particular clinical and health importance. These studies can make a significant contribution to the early identification of carriers, the control of the spread of resistant bacteria, and the development of effective preventive and therapeutic strategies.

This study aimed to investigate the nasal colonization pattern of diabetic and non-diabetic individuals with Gram-positive (*Staphylococcus aureus*) and Gram-negative (Enterobacteriaceae) bacteria. Phenotypic characteristics such as antibiotic resistance pattern and biofilm formation ability were studied. Moreover, the presence of resistance genes, including mecA (in *Staphylococcus aureus* isolates) and *bla*_CTX_, *bla*_SHV_, and *bla*_TEM_ (in Enterobacteriaceae isolates) was also evaluated.

## Materials and methods

### Study population and clinical data collection

The study participants were selected from individuals referred to the Endocrine and Metabolism Research Center (EMRC) in Isfahan, Iran. All participants gave written informed consent before their inclusion. The criteria for eligibility required diabetes patients to be at least 18 years old, have had diabetes for more than five years, have an HbA1c level of 8 mmol/L or higher, and not have diabetic foot ulcers [9]. For non-diabetes participants, the criteria included being at least 18 years old and having no history of diabetes. A total of 300 individuals were recruited, with 150 diabetes patients compared to 150 age- and sex-matched non-diabetes controls. Participants who had used antibiotics within the last three weeks had significant wounds, had acute diseases or infections, or had other private reasons for exclusion were excluded from the study.

### Data collection and processing

After obtaining informed consent, demographic and medical history information was obtained from the participants using a structured checklist. Then, nasal swab sampling was done using a sterile swab moistened with normal saline into each participant’s anterior nostrils, and the swab was used five times. For each specimen, we sample both nostrils sequentially using the same swab. Each swab was transported to the laboratory immediately after sampling, cultured on sheep blood agar (SBA), mannitol salt agar, and Eosin-methylene blue (EMB), and incubated for 24–48 h at 37 °C.

We then took all samples to be screened for *S. aureus* by colony morphology, Gram staining, catalase test, deoxyribonuclease test, and coagulase test. In addition, colonies on EMB plates were screened for Enterobacteriaceae by Gram staining, and biochemical tests including Triple Sugar Iron Agar (TSI), Citrate, SIM, MR, VP, Urea Agar, and PAD. The Zidet brand kit (ZiPars Company, Iran) was used for confirmatory identification of the isolates [10, 11].

### Antibiotic susceptibility test

All *S. aureus* isolates were assessed for susceptibility to a panel of 7 antibiotics, including cefoxitin, ciprofloxacin, clindamycin, erythromycin, gentamycin, tetracycline, and trimethoprim-sulfamethoxazole. The Enterobacteriaceae isolates were also assessed for antimicrobial susceptibility test to a panel of 7 antibiotics, including Amoxicillin, Ceftazidime, Ciprofloxacin, Trimethoprim-Sulfamethoxazole, Amikacin, Cefotaxime, and Cefixime. The Kirby–Bauer disk diffusion method was used to test susceptibility to all antibiotics, and diameter interpretations were based on the protocol of the Clinical and Laboratory Standards Institute guidelines (CLSI 2024) [10]. Strains were classified as multidrug-resistant (MDR) if they were non-susceptible to at least one agent in three or more antimicrobial categories [12].

### Phenotypic identification of MRSA isolates

All *S. aureus* strains were tested to identify MRSA. Those *S. aureus* strains that were positive for the *mecA* gene and/or resistance to cefoxitin were identified as MRSA. Those *S. aureus* strains that were negative for the *mecA* gene and sensitive to cefoxitin were identified as methicillin-sensitive *S. aureus* (MSSA) [10].

### Phenotypic identification of ESBL

The isolates were screened for ESBL production by the double disk diffusion method with Mueller-Hinton agar plates (Ebresco, Iran) and disks containing 30 µg of cefotaxime (CTX) and ceftazidime (CAZ) with or without 10 µg of clavulanic acid (CA) as recommended by the CLSI. A strain was regarded as an ESBL producer if the inhibition zone diameter for CTX or CAZ combined with CA exceeded that of CTX or CAZ alone by 5 mm or more [13].

### Biofilm assay

The microtiter plate method was performed according to previously reported instructions. Briefly, 200 µL of bacterial suspension grown in Trypticase Soy Broth (TSB) medium containing 1% glucose and diluted 1:100 was transferred to the wells of a sterile, flat-bottomed polystyrene 96-well plate. As a negative control, 200 µL of TSB medium containing 1% glucose without bacterial suspension was used. Samples were incubated for 24 h at 37 °C, and all experiments were performed in triplicate. Then, each well was washed three times with sterile phosphate-buffered saline (PBS; pH 7.2). The fixation step was performed by adding 150 µL of methanol to each well and after 15 min plates were emptied and left to dry. Subsequently, the adherent biofilm layer was stained with crystal violet for 15 min at room temperature, followed by a series of washing steps. The plates were then air-dried and solubilized with 95% ethanol for 30 min. Finally, the optical absorbance (OD) of each well was measured at 570 nm, and the average absorbance values of the negative controls and samples were calculated. The results were interpreted according to the previous reports [14, 15]. *Staphylococcus epidermidis* ATCC 35,984 was used as the biofilm producer control strain.

### Molecular characterization

The bacterial isolates, identified as *S. aureus* by specific phenotypic and biochemical features, were further confirmed by PCR using specific primers for the *nuc* gene (Table 1) to amplify a 270 bp internal fragment.

**Table 1:** Primer sequences and expected size of the PCR product

Gene	Sequence (5′-3′)	Product size (bp)
*nuc*	F: 5’-GCGATTGATGGTGATACGGTT-3’	270
	R: 5’-AGCCAAGCCTTGACGAACTAAAGC-3’	
*mecA*	F: 5’-AACTGATCAATTTGATGAACAAG-3’	352
	R: 5’-ATCTGTTGGATATGCAAACTC-3’	
*bla*_SHV_	F: 5’-TGGGAAACGGAACTGAATGAG-3’	160
	R: 5’-TCGTCCACCATCCACTGCAG-3’	
*bla*_TEM_	F: 5’-CACCAGTCACAGAAAAGCATC-3’	164
	R: 5’-GTTAGCTCCTTCGGTCCTCC-3’	
bla_CTX_	F: 5’-CTACAGTACAGCGATAACGTG-3’	279
	R: 5-GGAATGGCGGTGTTTAACGTC-3’	

Regarding resistance genes, we detected *mecA* to confirm MRSA isolates. The Enterobacteriaceae isolates were further tested by multiplex polymerase chain reaction (PCR) for the presence of ESBL genes, including *bla*_TEM_, *bla*_SHV_, and *bla*_CTX,_ using the designed primer set presented in Table 1.

Bacterial DNA was extracted by boiling 1 mL of the overnight culture, as described by Parvin et al. Colony PCR was performed as following steps: the tip of a toothpick was gently touched to a colony on agar plates, mixed with 20 µl injection water, and placed into the bain-marie at 95 °C for 5 min. The mixture was then used as a template for the PCRs [16]. PCR mixture was prepared in a 20 µl final volume (DNA template 1 µl, each primer 1 µl, master mix buffer 10 µl). The PCR was performed in a personal Boecco thermocycler (Germany) with an initial 5-minute denaturation at 95 °C, followed by 30 cycles of annealing at 55 °C for 1 min, extension at 72 °C for 1 min, and denaturation at 95 °C for 1 min, followed by a final extension step of 72 °C for 5 min [17]. Appropriate controls were included in each PCR run. The PCR products were visualized by electrophoresis on a 1% agarose gel containing safe stain (0.01%). The DNA bands were photographed using a UV transilluminator [17].

### Statistical analysis

Statistical analyses were done using SPSS software for Windows (SPSS, Inc., Chicago, IL, USA, version 25). Descriptive data are reported as mean ± SD, median [IQR], or number (percent) as appropriate. Independent sample t-test, chi-square test, and Fisher’s exact test were used as appropriate. All hypothesis testing was two-tailed, and the level of significance was considered to be less than 0.05 in all tests.

## Results

### General characteristic

A total of 300 participants were included in the study. Of those, 150 were the diabetes population, and 150 were the non-diabetes population. There were 60 (40.0%) men and 90 (60.0%) women in the diabetes population, whereas there were 59 (39.3%) men and 91 (60.6%) women in the non-diabetes population. The mean ages were 59.1 ± 12.1 and 58.6 ± 13.1 years for the diabetic and non-diabetic population, respectively. Further details on clinical characteristics are listed in Table 2. Diabetic participants had an average diabetes duration of 10.6 ± 6.1 years, with durations varying from 2 to 30 years. Among the clinical characteristics, history of COVID-19 infection and history of antibiotic use were statistically significant between the two groups (Table 2).

**Table 2:** Characteristics of diabetes and non-diabetes participants in this study

Demographic and clinical characteristics	Diabetes (*n* = 150)	Non-diabetes (*n* = 150)	*p*-value
Age (years)	59.1 ± 12.1	58.6 ± 13.1	0.714
Gender F/M	90/60	91/59	0.906
Hospitalization	24 (16.0)	16 (10.7)	0.174
Background disease	32 (21.6)	28 (18.7)	0.525
Antibiotic use*	37 (24.8)	17 (11.3)	0.002
Covid-19	18 (12.1)	50 (33.3)	< 0.0001
S. aureus	28 (18.7)	19 (12.7)	0.153
MRSA (out of *S. aureus*)	12 (42.8)	2 (10.5)	0.029
MDR (out of *S. aureus*)	9 (32.1)	6 (31.6)	0.968
Enterobacteriaceae isolates	5 (3.33)	11 (7.33)	0.124

• Data presented as Number (Percent)

• *P*-values calculated by Chi-square or Fisher exact test

• * History of antibiotic use during the last six months

### Nasal colonization of *S. aureus*

Of the 150 diabetes and 150 non-diabetes participants, *S. aureus* was colonized in (28, 18.7%) and (19, 12.7%), from whom (12, 42.8%) and (2, 10.5%) were MRSA, respectively. There was a statistically significant difference between the two populations in MRSA nasal colonization.

No association was found between demographic (age and sex) and some clinical variables (hospitalization and background disease) and nasal colonization of *S. aureus* in all participants (all *p*-values > 0.05) (Table 2).

The highest proportion of antibiotic resistance in nasal colonization of *S. aureus* was to tetracycline, with 53.6% and 47.4% among diabetic and non-diabetic participants, respectively. The lowest frequency of antibiotic resistance in nasal *S. aureus* isolates was observed for gentamicin, with rates of 3.6% and 5.3% in diabetic and non-diabetic individuals, respectively (Table 3). Furthermore, there was no statistically significant difference in the occurrence of MDR *S. aureus* between the diabetic and non-diabetic groups. Using the microtiter plate method due to the negative control OD (OD cutoff = 0.064), the majority of *S. aureus* strains (87.0%, 13 in non-diabetes and 28 in diabetes) were weak biofilm producers (0.064 < ODs ≤ 0.128). In the non-diabetes group, six isolates were considered to be moderate biofilm producers (3, 6.5%) and non-adherent (3, 6.5%) (0.128 < ODs ≤ 0.256).

**Table 3:** Antibiotic resistance of *Staphylococcus aureus* nasal colonization

Antibiotics	Diabetic population(*n*=28)	Non-diabetic population(*n*=19)	*p*-value
Cefoxitin	9 (32.1)	2 (10.5)	0.159
Ciprofloxacin	10 (35.7)	6 (31.6)	0.769
Clindamycin	7 (25.0)	6 (31.6)	0.621
Erythromycin	8 (28.6)	7 (36.8)	0.551
Gentamycin	1 (3.6)	1 (5.3)	> 0.999
Tetracycline	15 (53.6)	9 (47.4)	0.676
Trimethoprim-sulfamethoxazol	6 (21.4)	2 (10.5)	0.445

• Data presented as Number (Percent)

• *P*-values calculated by Chi-square or Fisher's exact test

All *S. aureus* isolates, as determined by their specific phenotypic and biochemical features, harbored the *nuc* gene. Among the 12 MRSA isolates identified in the diabetic group, 8 isolates showed genotypic and phenotypic resistance, 3 isolates were only *mec*A positive, and one isolate was resistant to cefoxitin but lacked the *mec*A gene.

Table 4 displays antibiotic-resistant patterns and biofilm phenotype of *Staphylococcus aureus* nasal colonization in both diabetic and non-diabetic participants.

**Table 4:** Antibiotic-resistant patterns and biofilm phenotype of *Staphylococcus aureus* nasal colonization in diabetes and non-diabetes groups

Population	mecA	MRSA	MDR	Antibiotic resistance patterns	Biofilm phenotype
Diabetes(*n* = 28)	-	-	+	FOX-CP-CD-E-TE	W
	+	+	-	FOX	W
	+	+	+	CP-CD-E-TE	W
	-	-	-	SXT	W
	+	+	-	-	W
	+	+	-	-	W
	-	-	+	CP- SXT-TE	W
	-	+	-	FOX	W
	+	+	+	CP-CD-E-SXT-TE	W
	-	-	-	SXT	W
	-	-	-	-	W
	+	+	-	TE	W
	+	+	-	TE	W
	-	-	-	-	W
	-	-	-	-	W
	-	-	-	-	W
	-	-	+	CP-CD-E- SXT-TE	W
	-	-	-	-	W
	-	-	-	-	W
	-	-	+	CP-CD-E-TE	W
	+	+	-	CD- E	W
	+	+	+	FOX- CP-GM- E-SXT-TE	W
	+	+	-	FOX- TE	W
	-	-	+	FOX- CP- TE	W
	-	-	-	CP- TE	W
	-	-	+	FOX- CP-CD-E-TE	W
	+	+	-	-	W
	-	-	-	FOX- TE	W
Non-diabetes (*n* = 19)	-	-	-	TE	W
	-	-	-	-	W
	-	-	+	FOX- CP- GM- CD- E- TE	W
	-	-	-	-	W
	-	-	-	TE	W
	+	+	-	FOX	W
	-	-	-	-	N
	-	-	+	CP- CD- E- SXT- TE	N
	-	-	-	E	W
	+	+	+	Fox- CP- CD- E- TE	W
	-	-	-	TE	N
	-	-	+	CP- CD- E- TE	W
	-	-	+	CP- CD- E- TE	W
	-	-	+	FOX-CP- CD- E- TE	W
	-	-	-	-	W
	-	-	-	-	M
	-	-	-	-	W
	-	-	-	SXT	M
	-	-	-	-	M

*Abbreviation*: *MRSA *Methicillin-resistant *Staphylococcus aureus*, *MDR *Multi-drug resistant, *CP *Ciprofloxacin, *SXT *Trimethoprim-sulfamethoxazole, *FOX *Cefoxitin, *CD *Clindamycin, *E* Erythromycin, *TE *Tetracycline, *GM *Gentamycin, *W *weakly biofilm producer, *M *moderate biofilm producer, *N *non-adherent

### Nasal colonization of Enterobacteriaceae

Out of 300 participants, 5 (3.3%) and 11 (7.3%) were colonized with Enterobacteriaceae isolates in the diabetes and non-diabetes groups, respectively. The identification of bacteria was isolated by biochemical tests and the API kit, which was mainly related to *Escherichia coli* (9/300, 3.0%) and *Enterobacter* spp. (7/300, 2.33%). No species from the family Enterobacteriaceae were isolated from participants who tested positive for *S. aureus*.

The highest proportion of antibiotic resistance in Enterobacteriaceae isolates among diabetes participants was cefixime (50%) and trimethoprim-sulfamethoxazole (50%). Antibiotic resistance to the other antibiotics is also presented in Table 5.

**Table 5:** Antibiotic resistance of Enterobacteriaceae nasal colonization

Antibiotics		Diabetic population(*n*=5)		Non-diabetic population(*n*=11)		*p*-value
Ceftazidime		1 (20.0)		1 (9.1)		0.554
Amikacin		0		1 (9.1)		0.500
Amoxicillin		2 (40.0)		2 (18.2)		0.366
Ciprofloxacin		1 (20.0)		2 (18.2)		0.934
Trimethoprim-Sulfamethoxazole		3 (60.0)		1 (9.1)		0.035
Cefixime		2 (40.0)		3 (27.3)		0.623
Cefotaxime		0		3 (27.3)		0.209

• Data presented as Number (Percent)

• *P*-values calculated by Chi-square or Fisher's exact test

In the biofilm formation assay, 43.8%, 37.5%, 6.2%, and 12.5% of the isolates were classified as non-adherent, weak, moderate, and strong biofilm producers, respectively. While phenotypic testing using the double-disk diffusion method did not identify any ESBL producers, 40.0% of isolates from diabetic participants and 45.4% from non-diabetic participants were positive for all three beta-lactamase genes (*bla*_CTX_, *bla*_TEM_, *and bla*_SHV_). Genotypic and phenotypic characteristics of Enterobacteriaceae nasal colonization in diabetes and non-diabetes groups are shown in Table 6.

**Table 6:** Genotypic and phenotypic patterns of Enterobacteriaceae nasal colonization in diabetes and non-diabetes groups

Population		Biofilm phenotype	Antibiotic resistance patterns	MDR	*bla*HSV	*bla*CTX	*bla*TEM
Diabetes(*n*= 5)		W	CAZ, SXT, CFM	-	+	+	+
		W	-	-	+	-	+
		S	CFM, AMC, CP, SXT	+	+	+	+
		N	CAZ	-	-	+	+
		N	AMC, SXT		-	-	+
Non-diabetes(*n*= 11)		W	-	-	+	+	+
		W	CTX	-	-	-	+
		N	CTX	-	-	+	+
		S	CTX, AN, AMC, CP	+	+	-	+
		W	CFM, SXT	-	+	+	+
		W	CFM	-	+	+	+
		M	-	-	+	-	+
		N	CFM	-	-	-	+
		N	CFM	-	+	+	+
		N	CP	-	+	+	+
		N	AMC	-	+	-	+

*Abbreviation*: *N *non-adherent, *W *weakly biofilm producer, *M *moderate, *S *strong biofilm producer, *CAZ *Ceftazidime, *SXT *Trimethoprim-sulfamethoxazole, *CFM *Cefepime, *CP* Ciprofloxacin, *AMC *Co-amoxiclav, *CTX *Cefotaxime, *AN *Amikacin, *MDR *Multi-drug resistant

## Discussion

The present study aims to enhance our current understanding of nasal colonization patterns and their phenotypic characteristics among diabetic outpatients and non-diabetic individuals.

### Nasal colonization of *S. aureus*

The proportion of nasal colonization of *S. aureus* (18.7%, 28/150) among the diabetic participants in this study was higher than that reported in diabetic populations in community settings in China (8.70%, 46/529) [10] and among type 2 diabetes outpatients in China (10.31%, 43/417) [18], but lower than that observed among diabetic participants in the US (28.32%, 286/1010) [19], diabetic patients in Australia (39.09%, 258/660) [11], diabetic outpatients in Turkey (41.78%, 127/304) [20], hospitalized diabetic patients in China (20.50%, 41/200) [21], hospitalized diabetic patients in India (56.67%, 34/60) [3], and long-term hemodialysis patients with type 2 diabetes in Saudi Arabia (72.41%, 42/58) [22].

The proportion of MRSA (8.0%, 12/150) among the diabetes participants in this study was higher than diabetes participants in the US (1.09%, 11/1010) [18], diabetes patients in Australia (1.21%, 8/660) [11], community-based diabetes population in China (4.16%, 22/529) [10], those of type 2 diabetes patients in China (5.28%, 22/417) [19], hospitalized diabetic patients in China (0.50%, 1/ 200) [20], and lower than the diabetic outpatient population in Turkey (9.87%, 30/304) [21] and long-term hemodialysis type 2 diabetes patients in Saudi Arabia (18.97%, 11/58) [22].

Although MRSA isolates lacking the *mec*A gene are rare, it is possible that the observed phenotypic resistance is due to the presence of the *mec*C gene, which is what is called *mec*A-negative MRSA [23–25].

Fortunately, contrary to many other studies [10, 18]High antibiotic resistance was not observed except for tetracycline (nearly 50%) in both groups. This might be a result of the widespread use of this antibiotic among people. Despite other studies reporting relatively high resistance to erythromycin above 50% [10, 18]. The results of the present study showed lower resistance, 28.6% and 36.8% in the diabetes and non-diabetes groups, respectively.

Although the proportion of MDR *S. aureus* strains in the diabetes population (32.1%) was not statistically different from the non-diabetes population (31.6%), this relatively high proportion should be noticed by the healthcare system to reasonably utilize antibiotics.

### Nasal colonization of enterobacteriaceae

The proportion of nasal colonization with *Enterobacteriaceae* in this study (5.3%, 16/300) was considerably lower than that reported in a hospital in Madagascar (53.0%, 816/1541) and the rate found in the general population in Germany (33.4%), and higher than type 2 diabetic patients in Poland (2.2%, 2/88) [26].

The proportion of multidrug-resistant (MDR) Enterobacteriaceae isolates among participants in our study (12.5%, 6 out of 48) was higher than that in the German population (0%, 0/628), where antibiotic resistance was very low, and no ESBL-producing Klebsiella or Escherichia coli isolates were detected. This indicates that the MDR rate in the German population was zero [27]. However, the MDR rate in our study is still lower than expected in regions with poor hygiene or higher antibiotic misuse.

Overall, while the nasal cavity is not considered a major reservoir for Enterobacteriaceae and the detected prevalence of nasal colonization in our study population (5.3%, 16/300) was relatively low, the presence of multidrug-resistant (MDR) strains (12.5%, 2/16) underscores the importance of ongoing monitoring in both diabetic and non-diabetic groups.

### Limitations and strengths

The greatest strength of this study is that it simultaneously investigates of nasal colonization pattern with *S. aureus* and Enterobacteriaceae isolates in diabetic and non-diabetic groups. There were also several limitations to the present study. First, we did not have a large sample size because of limited financial support. Second, we did not follow up on the outcomes of *S. aureus* and MRSA nasal colonization among the diabetes population.

## Conclusions

In conclusion, this study is the first to concurrently investigate nasal colonization patterns of *Staphylococcus aureus* and *Enterobacteriaceae* isolates, revealing a notably higher prevalence of MRSA among diabetic individuals compared to their non-diabetic counterparts. Moreover, although the nasal cavity is not a typical reservoir for *Enterobacteriaceae*, the observed moderate rate of MDR carriage emphasizes the necessity of ongoing surveillance in both diabetic and non-diabetic populations. 

## Supplementary Information

Supplementary Material 1.

Supplementary Material 2.

Supplementary Material 3.

Supplementary Material 4.

Supplementary Material 5.

## Data Availability

The datasets used and/or analyzed during the current study are available from the corresponding author on reasonable request.

## Abbreviations

DFIs: Diabetic foot infections
MRSA: Methicillin-resistant *Staphylococcus aureus*
MSSA: Methicillin-sensitive *S. aureus*
ESBL: Extended-spectrum beta-lactamases
EMRC: Endocrine and Metabolism Research Center
SBA: Sheep blood agar
EMB: Eosin-methylene blue
TSI: Triple Sugar Iron Agar
CLSI: Clinical and Laboratory Standards Institute guidelines
MDR: Multidrug resistant
CTX: Cefotaxime
CAZ: Ceftazidime
CA: Clavulanic acid
TSB: Trypticase Soy Broth
PBS: Phosphate-buffered saline
OD: Optical absorbance
PCR: Polymerase chain reaction
