# Supplementary material for: The spectrum of nasal colonization: frequency and resistant patterns in diabetes versus non-diabetes population
Source: BMC Microbiol. 2026 Feb 4;26:201. doi: 10.1186/s12866-026-04751-z (PMC12958542; doi:10.1186/s12866-026-04751-z)
Supplement: Supplementary file 1 — Supplementary Material 1. [file 12866_2026_4751_MOESM1_ESM.docx]

**Supplementary file**

| Table S1. Genotypic and phenotypic patterns of Enterobacteriaceae nasal colonization in diabetes and non-diabetes groups. | | | | | | | |
| --- | --- | --- | --- | --- | --- | --- | --- |
| Population | | **Biofilm phenotype** | **Antibiotic resistance patterns** | **MDR** | ***bla _HSV_*** | ***bla _CTX_*** | ***bla _TEM_*** |
| Diabetes  (n= 5) | | **W** | CAZ, SXT, CFM | **-** | **+** | **+** | **+** |
|  |  | **W** | - | **-** | **+** | **-** | **+** |
|  |  | **S** | CFM, AMC, CP, SXT | **+** | **+** | **+** | **+** |
|  |  | **N** | CAZ | **-** | **-** | **+** | **+** |
|  |  | **N** | AMC, SXT |  | **-** | **-** | **+** |
| Non-diabetes  (n= 11) | | **W** | - | **-** | **+** | **+** | **+** |
|  |  | **W** | CTX | **-** | **-** | **-** | **+** |
|  |  | **N** | CTX | **-** | **-** | **+** | **+** |
|  |  | **S** | CTX, AN, AMC, CP | **+** | **+** | **-** | **+** |
|  |  | **W** | CFM, SXT | **-** | **+** | **+** | **+** |
|  |  | **W** | CFM | **-** | **+** | **+** | **+** |
|  |  | **M** | - | **-** | **+** | **-** | **+** |
|  |  | **N** | CFM | **-** | **-** | **-** | **+** |
|  |  | **N** | CFM | **-** | **+** | **+** | **+** |
|  |  | **N** | CP | **-** | **+** | **+** | **+** |
|  |  | **N** | AMC | **-** | **+** | **-** | **+** |
| Abbreviation: N: non-adherent, W: weakly biofilm producer, M: moderate, S: strong biofilm producer, biofilm producer, CAZ: Ceftazidime, SXT: Trimethoprim-sulfamethoxazole, CFM: Cefepime, CP: Ciprofloxacin, AMC: Co-amoxiclav, CTX: Cefotaxime, AN: Amikacin, MDR: Multi-drug resistant. | | | | | | | |
