# Supplementary material for: The spectrum of nasal colonization: frequency and resistant patterns in diabetes versus non-diabetes population
Source: BMC Microbiol. 2026 Feb 4;26:201. doi: 10.1186/s12866-026-04751-z (PMC12958542; doi:10.1186/s12866-026-04751-z)
Supplement: Supplementary file 2 — Supplementary Material 2. [file 12866_2026_4751_MOESM2_ESM.pdf]

SmallSEOTools

Plagiarism Detection Report by SmallSEOTOOLS

0%

Plagiarism	0%	Partial Match	0%
Exact Match	0%	Unique	100%

Scan details

Total Words	Total Characters	Plagiarized Sentences	Unique Sentences
530	3739	0	23 (100%)

#1 100% Unique

The nasal cavity and nasopharynx are among the body's first points of contact with the external environment, and as part of the upper respiratory tract, they play a crucial role in the initial defense against pathogens. This area hosts diverse microbial communities that include symbiotic, opportunistic, and sometimes potential pathogens [1]. The composition of the nasal microbiota is influenced by several factors, including age, environmental conditions, the individual's immune status, and underlying diseases. Patients with diabetes are at a higher risk of colonization and infection with opportunistic microorganisms due to impaired immune function, changes in the composition of the body's natural flora, and increased exposure to healthcare facilities. Among the nasal colonizing bacteria, S. aureus is the focus of clinical research, particularly due to its ability to transform from an asymptomatic colonizer to an invasive pathogen [2].

Staphylococcus aureus is one of the most important human pathogens and a leading cause of severe infections associated with high mortality, morbidity, and significant healthcare costs. Its wide array of virulence factors enables it to cause multiple clinical manifestations such as bacteremia, endocarditis, osteomyelitis, and infections of the skin, soft tissues, bones, lungs, and medical devices [3]. According to a systematic review and meta-analysis, mortality rates from S. aureus bacteremia are 18.1% at one month, 27.0% at three months, and 30.2% at one year (4). S. aureus is also frequently isolated from diabetic foot infections (DFIs) worldwide. A recent systematic review in Iran reported that S. aureus accounts for 24.29% of bacterial isolates from DFIs, with 55% of them being methicillin-resistant (MRSA) [4]. Moreover, up to 30% of the general population may carry S. aureus in their nasal passages. Widespread use of antibiotics has led to the emergence of resistant strains such as MRSA, which has significantly increased morbidity and mortality in both hospital and community settings. Occasionally, nasal colonization by S. aureus may progress to serious opportunistic infections [5].

On the other hand, although gram-negative bacteria are not usually recognized as the dominant nasal flora, recent studies have shown that gram-negative bacilli such as Klebsiella spp., Escherichia coli, Enterobacter spp., and Pseudomonas spp. can also lead to nasal colonization in certain circumstances, including hospitalized patients, people with diabetes, or those with a history of antibiotic use. The importance of these bacteria is doubled when they are associated with multiple drug resistance, especially the production of extended-spectrum beta-lactamases (ESBL) [6]. Considering the increasing prevalence of diabetes worldwide and the key role of nasal bacteria in nosocomial and community-acquired infections, the study of colonization patterns of Gram-positive and Gram-negative bacteria in the nose of diabetic patients is of particular clinical and health importance. These studies can make a significant contribution to the early identification of carriers, the control of the spread of resistant bacteria, and the development of effective preventive and therapeutic strategies.

This study aimed to investigate the nasal colonization pattern of diabetic and non-diabetic individuals with Gram-positive (Staphylococcus aureus) and Gram-negative (Enterobacteriaceae) bacteria. Phenotypic characteristics such as antibiotic resistance pattern and biofilm formation ability were studied. Moreover, the presence of resistance genes, including mecA (in Staphylococcus aureus isolates) and bla CTX, bla SHV, and bla TEM (in Enterobacteriaceae isolates) was also evaluated.
