# Supplementary material for: The spectrum of nasal colonization: frequency and resistant patterns in diabetes versus non-diabetes population
Source: BMC Microbiol. 2026 Feb 4;26:201. doi: 10.1186/s12866-026-04751-z (PMC12958542; doi:10.1186/s12866-026-04751-z)
Supplement: Supplementary file 3 — Supplementary Material 3. [file 12866_2026_4751_MOESM3_ESM.pdf]

SmallSEOTools

Plagiarism Detection Report by SmallSEOTOOLS

0%

Plagiarism	0%	Partial Match	0%
Exact Match	0%	Unique	100%

Scan details

Total Words	Total Characters	Plagiarized Sentences	Unique Sentences
725	4845	0	34 (100%)

#1 100% Unique

The study participants were selected from individuals referred to the Endocrine and Metabolism Research Center (EMRC) in Isfahan, Iran. All participants gave written informed consent before their inclusion. The criteria for eligibility required diabetes patients to be at least 18 years old, have had diabetes for more than five years, have an HbA1c level of 8 mmol/L or higher, and not have diabetic foot ulcers [7]. For non-diabetes participants, the criteria included being at least 18 years old and having no history of diabetes. A total of 300 individuals were recruited, with 150 diabetes patients compared to 150 age- and sex-matched non-diabetes controls. Participants who had used antibiotics within the last three weeks had significant wounds, had acute diseases or infections, or had other private reasons for exclusion were excluded from the study.

Data Collection and Processing

After obtaining informed consent, demographic and medical history information was obtained from the participants using a structured checklist. Then, nasal swab sampling was done using a sterile swab moistened with normal saline into each participant's anterior nostrils, and the swab was used five times. For each specimen, we sample both nostrils sequentially using the same swab. Each swab was transported to the laboratory immediately after sampling, cultured on sheep blood agar (SBA), mannitol salt agar, and Eosin-methylene blue (EMB), and incubated for 24-48 h at 37°C.

We then took all samples to be screened for S. aureus by colony morphology, Gram staining, catalase test, deoxyribonuclease test, and coagulase test. In addition, colonies on EMB plates were screened for Enterobacteriaceae by Gram staining, and biochemical tests including Triple Sugar Iron Agar (TSI), Citrate, SIM, MR, VP, Urea Agar, and PAD. The Zidet brand kit (ZiPars Company, Iran) was used for confirmatory identification of the isolates [8, 9].

Antibiotic Susceptibility Test

All S. aureus isolates were assessed for susceptibility to a panel of 7 antibiotics, including cefoxitin, ciprofloxacin, clindamycin, erythromycin, gentamycin, tetracycline, and trimethoprim-sulfamethoxazole. The Enterobacteriaceae isolates were also assessed for antimicrobial susceptibility test to a panel of 7 antibiotics, including Amoxicillin, Ceftazidime, Ciprofloxacin, Trimethoprim-Sulfamethoxazole, Amikacin, Cefotaxime, and Cefixime. The Kirby–Bauer disk diffusion method was used to test susceptibility to all antibiotics, and diameter interpretations were based on the protocol of the Clinical and Laboratory Standards Institute guidelines (CLSI 2024) [10]. Strains were classified as multidrug-resistant (MDR) if they were

Phenotypic identification of MRSA isolates

All S. aureus strains were tested to identify MRSA. Those S. aureus strains that were positive for the mecA gene and/or resistance to cefoxitin were identified as MRSA. Those S. aureus strains that were negative for the mecA gene and sensitive to cefoxitin were identified as methicillin-sensitive S. aureus (MSSA) [10].

Phenotypic identification of ESBL

The isolates were screened for ESBL production by the double disk diffusion method with Mueller-Hinton agar plates (Ebresco, Iran) and disks containing 30 µg of cefotaxime (CTX) and ceftazidime (CAZ) with or without 10 µg of clavulanic acid (CA) as recommended by the CLSI. A strain was regarded as an ESBL producer if the inhibition zone diameter for CTX or CAZ combined with CA exceeded that of CTX or CAZ alone by 5 mm or more [12].

Biofilm assay

The microtiter plate method was performed according to previously reported instructions. Briefly, 200 µL of bacterial suspension grown in Trypticase Soy Broth (TSB) medium containing 1% glucose and diluted 1:100 was transferred to the wells of a sterile, flat-bottomed polystyrene 96-well plate. As a negative control, 200 µL of TSB medium containing 1% glucose without bacterial suspension was used. Samples were incubated for 24 hours at 37°C, and all experiments were performed in triplicate. Then, each well was washed three times with sterile phosphate-buffered saline (PBS; pH 7.2). The fixation step was performed by adding 150 µL of methanol to each well. Subsequently, the adherent biofilm layer was stained with crystal violet for 15 minutes at room temperature, followed by a series of washing steps. The plates were then air-dried and solubilized with 95% ethanol for 30 minutes. Finally, the optical absorbance (OD) of each well was measured at 570 nm, and the average absorbance values of the negative controls and samples were calculated. Interpretation of results was described before by Moghadam et al. [13, 14]. Staphylococcus epidermidis ATCC 35984 was used as the biofilm producer control strain
