# Supplementary material for: The spectrum of nasal colonization: frequency and resistant patterns in diabetes versus non-diabetes population
Source: BMC Microbiol. 2026 Feb 4;26:201. doi: 10.1186/s12866-026-04751-z (PMC12958542; doi:10.1186/s12866-026-04751-z)
Supplement: Supplementary file 4 — Supplementary Material 4. [file 12866_2026_4751_MOESM4_ESM.pdf]

Plagiarism Detection Report by SmallSEOTOOLS

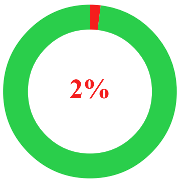

Plagiarism	2%	Partial Match	0%
Exact Match	2%	Unique	98%

Scan details

Total Words	Total Characters	Plagiarized Sentences	Unique Sentences
**893**	**5856**	**0.9**	**44.1 (98%)**

Plagiarism Results: (1)

#1 2% Similar https://pubmed.ncbi.nlm.nih.gov/30739927

A total of 300 participants were included in the study.
