# Supplementary material for: The spectrum of nasal colonization: frequency and resistant patterns in diabetes versus non-diabetes population
Source: BMC Microbiol. 2026 Feb 4;26:201. doi: 10.1186/s12866-026-04751-z (PMC12958542; doi:10.1186/s12866-026-04751-z)
Supplement: Supplementary file 5 — Supplementary Material 5. [file 12866_2026_4751_MOESM5_ESM.pdf]

Plagiarism Detection Report by SmallSEOTOOLS

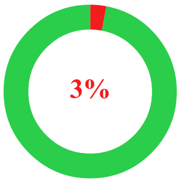

Plagiarism	3%	Partial Match	0%
Exact Match	3%	Unique	97%

Scan details

Total Words	Total Characters	Plagiarized Sentences	Unique Sentences
**694**	**4829**	**0.87**	**28.13 (97%)**

Plagiarism Results: (1)

#1 3% Similar https://pmc.ncbi.nlm.nih.gov/articles/PMC5415482

aureus and MRSA nasal colonization among the diabetes population.
